# Editorial: The adaptive role of musicality in human evolution

**DOI:** 10.3389/fpsyg.2024.1419170

**Published:** 2024-05-15

**Authors:** Nicholas Bannan, Robin I. M. Dunbar, Alan R. Harvey, Piotr Podlipniak

**Affiliations:** ^1^The Conservatorium of Music, University of Western Australia, Perth, WA, Australia; ^2^Department of Experimental Psychology, University of Oxford, Radcliffe Observatory Quarter, Oxford, United Kingdom; ^3^School of Human Sciences and Conservatorium of Music, The University of Western Australia, Crawley, WA, Australia; ^4^Perron Institute for Neurological and Translational Science, Nedlands, WA, Australia; ^5^Department of Musicology, Adam Mickiewicz University, Poznań, Poland

**Keywords:** musicality, aural perception, language, song, evolution, culture, communication, harmonicity

## Introduction

Over the last 40 years or so, interest has re-emerged in the topic of human musical behavior and its evolution, represented both by monographs (e.g., Wallin, 1991; Mithen, 2005; Tomlinson, 2015; Harvey, 2017) and several compilations (Wallin et al., 2000; Bannan, 2012; Nikolsky and Perlovsky, 2020). In recent years, publications on this topic have increased in number and breadth. So: why publish yet another Research Topic in this field?

Our focus in devising the topic of this Research Topic was to encourage exploration of the acute and specialized nature of human aural perception and its evolved role in the capacity for music and language. Specifically, we aimed to encourage greater precision in accounts of how musical abilities are acquired and transmitted through vocal learning in relation to speech acquisition and musical expertise.

A key development, on which modern human vocal abilities depend, was the achievement of a feedback loop between the perception and production of vocal sound that allows precise matching, imitation, and meaningful variation, of the acoustic properties: fundamental frequency, duration (and its role in rhythm), amplitude, and timbral spectrum. These are the four specific properties that influence our sensations of sound. They play parallel and complementary roles in both language and vocal music, represented in blended synchrony such as unison song and collective speech; as well as temporally organized sequences such as call-and-response and polyphony.

This Research Topic comprises articles stimulated by this description of the topic, presenting studies that variously examine the evolution of these universal abilities. Fields represented include animal behavior, cultural evolution, and studies of the phenomenon of human musicality from a variety of perspectives.

## Locating this topic within the disciplinary frameworks that embrace it

The study of musicality depends upon developments within a wide range of disciplines and methods: anthropology, acoustics, neurology, and social psychology perhaps at the center; linguistics, archaeology, anatomy, animal behavior and audiology presenting essential extensions and bridges that expand their perspective. In focusing on the definition and exploration of musicality and its evolutionary foundations, this Research Topic has also attracted material from within musicology and ethnomusicology, presenting data that communicates musical phenomena in their own terms where much work in this field has proved generally descriptive or poorly informed.

## Convergent and divergent interpretations

The variety of approaches and disciplinary perspectives brought to bear on the issue of musicality represents a complex spectrum embracing several interacting axes. The “positioning” of research within these can both contribute clarification and provide innovative presentation of new data. This Issue adopted the stance that contributions need not represent agreement with one another, as if the editors were gatekeepers to interpretations that all would unanimously accept. Readers will judge for themselves the conviction with which they may respond to what the Issue contains, and how it may shape their own thinking on the topic.

Principal among interpretations that have led to divergent views have been:

Social Bonding (e.g., Dunbar, 2012; Harvey, 2017; Savage et al., 2021), in relation to Credibility of the Signal (e.g. Aitchison, 2000; Mehr et al., 2021); and the reconciliation of these in more recent models (Podlipniak, 2023; Bannan et al., in press; Bamford et al.);

Instinctive responses to harmonicity (e.g. Bannan et al., n.d.), in relation to scales and tunings as cultural preferences (Brown and Phillips);

Sexual dimorphism (e.g., Miller, 2011), in relation to abilities shared across genders (Fitch, 2006);

The universal and the unique: reconciling cultural contrasts (see Jacoby et al., 2019).

## The contribution made in this Research Topic and potential responses in future research

Comparison between animal and human behaviors defines and illustrates potential evolutionary patterns. Embracing a comprehensive discussion of the factors that may affect the responses of pet and laboratory animals to human communication, Seki reviews experiments that explore the role of rhythmic synchronization to a musical beat in vocal production learners. If the cockatiels studied can sing imitations of human music in synchrony with a playback of the melody, how would such an ability have evolved in a flocking species compared to the beat perception conferred by human collective locomotion? Tracing the stages by which modern human abilities emerged, Dunbar refines and re-presents the features of the social bonding mechanism that he places at the root of the musicality mosaic.

Bamford et al. revisit Darwin' account of the origins of human vocalization in sexual attraction, exploring a model for the continuing link between romantic love and musical interaction that reconciles social bonding with credible signaling strategies. By contrast, Jordania explores the role of musicality and other early human biological and behavioral traits as components of protection against predators that contributed to survival. In the research report of Chittar et al., close study of the singing behaviors of five hunter-gatherer women in the Republic of Congo tested the application of the credible signaling hypothesis. Amongst their findings were that group singing was not motivated by predation avoidance, but that carrying an infant while foraging correlates strongly with singing activity. The multi-sensory links between touch and infant-directed song (c.f. Dunbar) within the combined social and working context suggests a cultural influence in the establishment of parent-infant bonding.

Brown and Phillips focus on the nature of musical organization and its acquisition. Their article argues for the culturally-derived origin and transmission of tuning systems (e.g., musical scales) in contrast to the interval ratios associated with Pythagorean theory.

Jan defines and investigates the nature of musical memetics and their operation within and between repertoire examples, illustrating an evolutionary process that remains at work. Velička speculates on the stylistic features of a Lithuanian vocal repertoire that suggest ancient, prelinguistic origins.

This assembly of research reports and reviews evokes future directions in which research needs to further clarify the nature and practice of human musical behavior (see also Yurdum et al., 2023). Chittar et al. exemplify the potential of detailed data collection in non-WEIRD populations, in which further work is essential in order to ensure that a full understanding of the function and transmission of music is acquired.

## Author contributions

NB: Writing – original draft, Writing – review & editing. RD: Writing – original draft, Writing – review & editing. AH: Writing – original draft, Writing – review & editing. PP: Writing – original draft, Writing – review & editing.

## References

[B1] Aitchison J. (2000). The Seeds of Speech: Language Origin and Evolution. Cambridge: Cambridge University Press.

[B2] Bannan N. (2012). Music, Language, and Human Evolution. Oxford: Oxford University Press.

[B3] Bannan N. Dunbar R. I. M. Bamford J. S. (in press). The evolution of gender dimorphism in the human voice: the role of octave equivalence. Curr. Anthropol.

[B4] Bannan N. Dunbar R. I. M. Harvey A. R. Podlipniak P. (n.d.). Acoustic processing the origin of human vocal communication (in preparation).

[B5] Dunbar R. I. (2012). “On the evolutionary function of song and dance,” in Music, Language, and Human Evolution, ed. N. Bannan (Oxford: Oxford University Press), 201–214.

[B6] Fitch W. T. (2006). The biology and evolution of music: a comparative perspective. Cognition 100, 173–215. 10.1016/j.cognition.2005.11.009 16412411

[B7] Harvey A. R. (2017). Music, Evolution, and the Harmony of Souls. Oxford: Oxford University Press.

[B8] Jacoby N. Undurraga E. A. McPherson M. J. Valdés J. Ossandón T. McDermott J. H.. (2019). Universal and non-universal features of musical pitch perception revealed by singing. Curr. Biol. 29, 3229–3243. 10.1016/j.cub.2019.08.020 31543451 PMC9907018

[B9] Mehr S. A. Krasnow M. M. Bryant G. A. Hagen E. H. (2021). Origins of music in credible signaling. Behav. Brain Sci. 44:e60. 10.1017/S0140525X20000345 32843107 PMC7907251

[B10] Miller G. (2011). The Mating Mind: How Sexual Choice Shaped the Evolution of Human Nature. Milwaukee, WI: Anchor Press.

[B11] Mithen S. (2005). The Singing Neanderthals: The Origins of Music, Language, Mind and Body. London: Weidenfeld and Nicolson.

[B12] Nikolsky A. Perlovsky L. (2020). The evolution of music. Front. Psychol. 11:595517. 10.3389/fpsyg.2020.595517 33192939 PMC7655650

[B13] Podlipniak P. (2023). Free rider recognition—A missing link in the Baldwinian model of music evolution. Psychol. Music 51, 1397–1413. 10.1177/03057356221129319

[B14] Savage P. E. Loui P. Tarr B. Schachner A. Glowacki L. Mithen S.. (2021). Music as a coevolved system for social bonding. Behav. Brain Sci. 44:e59. 10.1017/S0140525X20000333 32814608

[B15] Tomlinson G. (2015). A Million Years of Music: The Emergence of Human Modernity. London: MIT Press.

[B16] Wallin N. L. (1991). Biomusicology: Neurophysiological, Neuropsychological, and Evolutionary Perspectives on the Origins and Purposes of Music. New York, NY: Pendragon Press.

[B17] Wallin N. L. Merker B. Brown S. (2000). The Origins of Music. London: MIT Press.

[B18] Yurdum L. Singh M. Glowacki L. Vardy T. Atkinson Q. D. Hilton C. B.. (2023). Universal interpretations of vocal music. Proc. Nat. Acad. Sci. 120:e2218593120. 10.1073/pnas.2218593120 37676911 PMC10500275

